# Acute Cardiovascular Events After COVID-19 in England in 2020: A Self-Controlled Case Series Study

**DOI:** 10.2147/CLEP.S421062

**Published:** 2023-09-01

**Authors:** Jennifer A Davidson, Amitava Banerjee, Helen Strongman, Emily Herrett, Liam Smeeth, Judith Breuer, Charlotte Warren-Gash

**Affiliations:** 1 Department of Non-Communicable Disease Epidemiology, London School of Hygiene and Tropical Medicine, London, UK; 2 Institute of Health Informatics, University College London, London, UK; 3 Department of Infection, Immunity and Inflammation, UCL Great Ormond Street Institute of Child Health, University College London, London, UK

**Keywords:** acute coronary syndrome, COVID-19, electronic health records, epidemiology, heart failure

## Abstract

**Purpose:**

To assess the risk of incident cardiovascular outcomes after COVID-19 by level of cardiovascular risk in waves one and two of the pandemic in England in 2020.

**Patients and methods:**

We conducted a self-controlled case-series study among adults aged 40–84 years with no pre-existing cardiovascular disease using linked data from the Clinical Practice Research Datalink. We generated season-adjusted incidence ratios (IRs) for first acute cardiovascular event after SARS-CoV-2 infection compared with baseline time before and >91 days after infection. We used composite and individual acute cardiovascular event outcomes including myocardial infarction, major ventricular arrhythmia, left ventricular heart failure, and ischemic stroke. We stratified by cardiovascular risk, using diagnosed hypertension and QRISK3 predicted risk, and by wave one and two of the pandemic.

**Results:**

We included 1762 individuals, 76.6% had a QRISK3 score ≥10% and 59.4% had hypertension. The risk of any cardiovascular event was elevated in the 1–7 days after infection (IR 7.14 [95% CI 6.06–8.41]) and, while the effect size tapered, the risk remained for 15–28 days after infection (1.74 [1.33–2.26]). Risks were similar for individual event type, differing by level of cardiovascular risk, and in wave one and two of the pandemic.

**Conclusion:**

SARS-CoV-2 infection is associated with early elevations in the risk of first acute cardiovascular event, across cardiovascular risk levels and in both wave one and two of the pandemic. Prevention of COVID-19 is important to avert cardiovascular complications.

## Graphical Abstract

**Figure:**
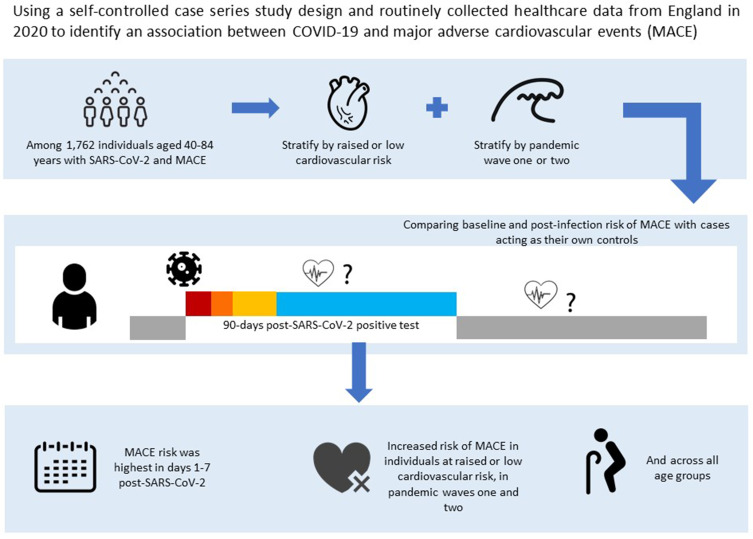

## Introduction

COVID-19 has been associated with cardiovascular complications,^1^ specifically self-controlled case series (SCCS) studies show elevated myocardial infarction (MI) and ischemic stroke incidences following infection.^2–4^ Results from Sweden found two-fold increases in first MI and ischemic stroke in the two weeks and one month, respectively, after COVID-19.^2^ A cohort analysis conducted using the same dataset supported the SCCS findings. A Danish SCCS analysis found three- and six-fold increases in first MI and stroke incidences, respectively, in the first month post-COVID-19,^3^ while a Scottish SCCS found five- and seven-fold increases in MI and ischemic stroke, respectively, within one-week post-infection.^4^ A UK cohort study found high MI and stroke risks in the first week after COVID-19, compared to no COVID-19, with risk remaining for months after infection.^5^ Similarly, results from a USA cohort study suggest long-term cardiovascular risk.^6^ Studies have also reported increased incidence of pulmonary embolism, deep vein thrombosis, myocarditis, acute heart failure, and arrhythmias following COVID-19.^4^,^5^,^7^

The mechanisms leading to COVID-19 triggered cardiovascular complications may include pro-inflammatory, pro-thrombotic and vasoconstrictive effects due to SARS-CoV-2 generated imbalances in ACE-2/RAS signalling.^8^ Biomarkers of cardiac injury, such as raised troponin, have been identified among COVID-19 patients with cardiovascular risk factors.^9^,^10^ Raised troponin has been associated with severe COVID-19 outcomes including mechanical ventilation and death, suggesting myocardial ischemia and injury may trigger severe outcomes.^11^ Individuals with raised cardiovascular risk may have altered cytokine profiles leading to systemic inflammation leading to a worsening of their disease when infected with SARS-CoV-2.^12^

Initial SCCS studies have shown a short-term increased incidence of cardiovascular complications following COVID-19,^2^,^3^ but there is limited England-specific analysis or understanding of how risk evolved over the pandemic. There is also uncertainty on the role background cardiovascular risk plays in COVID-19-related cardiovascular complications.^13^ In England, individuals with raised cardiovascular risk but without existing cardiovascular disease (CVD), were not considered “clinically vulnerable” to COVID-19, so not targeted or prioritized for any prevention measures.^14^

We used a SCCS analysis to investigate the association between COVID-19 and risk of a range of acute cardiovascular events, with stratification by underlying cardiovascular risk status and wave of the pandemic.

## Methods

### Data Sources

We used anonymized primary care data from the Clinical Practice Research Datalink (CPRD) Aurum January 2022 dataset (https://doi.org/10.48329/db7t-ay41), with linked patient-level secondary care data from the Hospital Episodes Statistics Admitted Patient Care (HES APC) database, COVID-19 Hospitalisations in England Surveillance System (CHESS) data, Second Generation Surveillance System (SGSS) SARS-CoV-2 PCR test results, and death registration data. CPRD Aurum is a growing dataset of longitudinal records from National Health Service (NHS) primary care general practitioners (GP), currently comprising >40 million individuals in England. The data collated include demographics, diagnoses, prescriptions, and immunizations. HES APC is a reimbursement dataset containing diagnosis and procedures collated from inpatient care at NHS hospitals in England. CHESS was set up at the start of the COVID-19 pandemic to provide timely surveillance of outcomes among hospitalized COVID-19 patients.

The CPRD Independent Scientific Advisory Committee (application 20_000135) and the London School of Hygiene and Tropical Medicine Ethics (LSHTM) Committee (application 22,717) approved the study. The CPRD provided data on relevant linked data for the study population.

### Study Design

We carried out a SCCS study which makes within-person comparisons. SCCS analysis only includes individuals with both the exposure and outcome. Individuals act as their own controls during periods of non-exposure.^15^ The main advantage of this design is the removal of confounding by fixed characteristics that vary between individuals.

SCCS analyses use conditional Poisson regression to model, and derive incidence ratios (IRs), the effect of a time-varying exposure on the outcome by comparing the incidence of events during exposed time with the incidence during unexposed (baseline) time.^15^ The SCCS method relies on several key assumptions.^15^ First, event recurrences must be independent ie, an event must not increase the probability of a further event. Second, an event should not impact subsequent exposure. Third, an event must not influence the end of the period of observation, which can be violated when the event increases the likelihood of mortality.

### Study Population and Follow-Up

CPRD-recorded patients aged 40–84 years (after which age all individuals are classified as having raised cardiovascular risk) who experienced their first acute cardiovascular event and had COVID-19 between 12 March 2020 (when daily reporting to CHESS was initiated)^16^ and 31 December 2020 were eligible for inclusion. Follow-up started at the latest of; age 40 years, 12 months post-GP registration, or 12 March 2020 and ended at the earliest date of; death, patient transfer out of GP practice, last data collection from the GP practice, or 31 December 2020. We excluded anyone with existing CVD – defined as heart disease (congenital or otherwise), heart failure, or cerebrovascular events – recorded in CPRD or HES APC. Individuals with CVD are more likely to have a further cardiovascular event, violating one of the key SCCS assumptions (as outlined in the study design section).

### Outcome

Our primary outcome was an acute cardiovascular event, defined as CPRD or HES APC recorded acute coronary syndrome (ACS) capturing MI and unstable angina, left ventricular heart failure, major ventricular arrhythmia, or ischemic stroke. Our secondary outcomes were each of the cardiovascular conditions separately. All codelists are published on LSHTM Data Compass.^17^

### Exposure

We defined COVID-19 as laboratory-confirmed SARS-CoV-2 using CPRD SGSS and CHESS data. Exposure date was the earliest specimen date within the follow-up period. In a secondary analysis, we redefined the exposure as clinically reported COVID-19 (recorded in CPRD or HES APC [ICD-10 codes U07.1 or U07.2 in primary diagnostic position]) without laboratory-confirmation, as early in the pandemic, COVID-19 testing was largely limited to individuals who were hospitalized.

### Statistical Analysis

We conducted all analyses in Stata (version 16).

We described key baseline characteristics of the whole study population and stratified by cardiovascular risk; age group (40–54, 55–64, 65–74 and 75–84), sex, COVID-19 associated hospital stay (defined by hospitalization due to COVID-19 based on HES APC ICD-10 codes U07.1 or U07.2 in primary diagnostic position or record in CHESS dataset), fatal events, and all deaths during follow-up.

We stratified our analyses by underlying cardiovascular risk using QRISK3 score and hypertension status. QRISK3 is a cardiovascular risk calculator used to estimate an individual’s ten-year likelihood of CVD, developed in QResearch, a UK EHR database. The score is widely used in UK primary care, as was the prior version QRISK2, and has been validated.^18^,^19^ The risk factors used in the score are age, sex, ethnicity, socioeconomic status, family history of coronary heart disease in a first degree relative aged <60 years, comorbid health conditions (diabetes, treated hypertension, rheumatoid arthritis, systemic lupus erythematosus, atrial fibrillation, chronic kidney disease stages 3–5, migraine, severe mental illness, HIV, erectile dysfunction) body mass index, systolic blood pressure reading and it’s variability, total cholesterol to high-density lipoprotein cholesterol ratio, smoking status, and corticosteroid treatment. We used our published Stata scripts^20^ to measure baseline risk factors using codes and measures recorded in patient CPRD records and applied the QRISK3 algorithm, published by the developers^21^ to assign individual scores. We assigned hypertension status using coded CPRD hypertension diagnoses within the five years before baseline or the most recent to baseline blood pressure (BP) reading with systolic BP of ≥140mmHg or diastolic BP of ≥90mmHg. We classified individuals with raised cardiovascular risk (hypertension or QRISK3 ≥10%) and low cardiovascular risk (no hypertension or QRISK3 <10%) at study entry.

**Figure 1:**
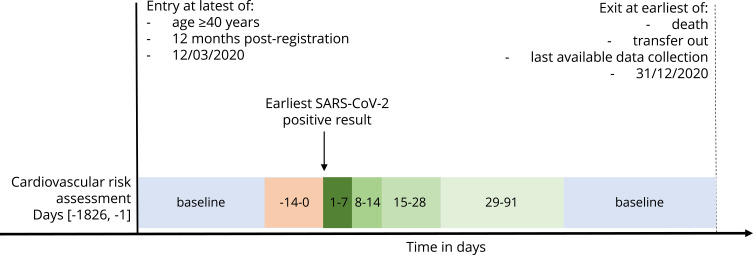
Study design with exposure and baseline periods.

**Notes**: Days 14–0 shown in orange were excluded from analysis and the time intervals shown in green are the strata of the exposed period used in analysis.

We compared the incidence of acute cardiovascular events in exposed periods (1–7, 8–14, 15–28, and 29–91 days) following COVID-19 with baseline (unexposed) periods (Figure 1, Graphical Abstract). We excluded the 14 days before the exposure date from the baseline because the temporal relationship between the infection and cardiovascular event cannot be determined. We used conditional Poisson regression to calculate IRs for acute cardiovascular events occurring within each exposed stratum compared with baseline. We adjusted for season split into the warmer months (April–September) and cooler months (October–March).

We further stratified results by the first UK COVID-19 wave (12 March–16 August) or second wave (17 August–31 December), during which different COVID-19 testing and clinical management strategies were in operation.^22^ Additional stratifying factors were age group and sex.

We repeated our initial analysis excluding fatal acute cardiovascular events. Acute cardiovascular events can result in death, violating the SCCS assumption that observation periods should end independent of event timing. We classified the fatal events as those for which the individual’s death date was ≤30 days after the event.

## Results

### Description of the Study Population

**Figure 2:**
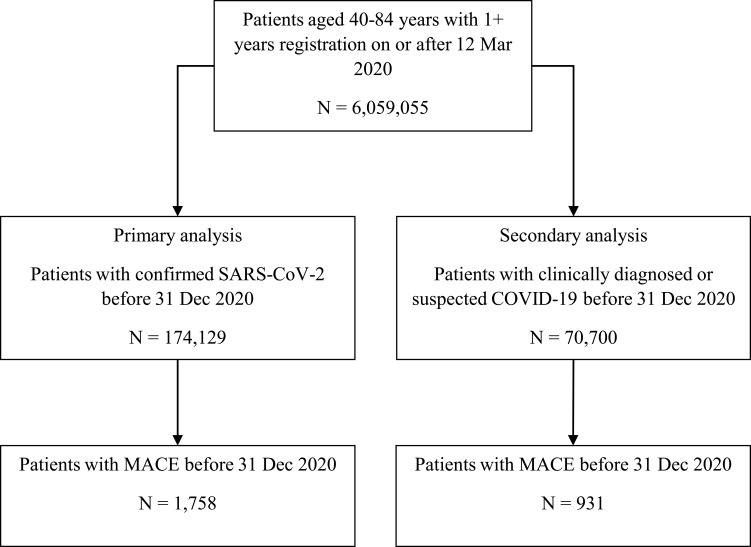
Study population flow chart.

We identified 1762 individuals with laboratory-confirmed SARS-CoV-2 and their first acute cardiovascular event in our study period (Figure 2), of which 569 (32.3%) were new onset of left ventricular heart failure, 565 (32.1%) were acute coronary syndrome, 401 (22.8%) were ischemic stroke and 227 (12.9%) were major ventricular arrhythmia. The study population characteristics are shown in Table 1. Most individuals had raised cardiovascular risk (QRISK3 ≥10%; 76.6%, n=1350 and hypertension; 59.4%, n=1047). There were more men (60.2%, n=1060) than women (39.8%, n=702). Nearly forty-percent (39.2%, n=691) of individuals were hospitalized due to their COVID-19. Over one-fifth (20.9%, n=369) of individuals died within 30 days of their cardiovascular event and more than one-quarter (26.0%, n=458) died during follow-up.

**Table 1:** Baseline Characteristics of Study Population

	All	QRISK3		Hypertension	
		Raised Risk	Low Risk	Raised Risk	Low Risk
	N=1762	N=1350	N=412	N=1047	N=715
Sex					
Women	702 (39.8%)	504 (37.3%)	198 (48.1%)	399 (38.1%)	303 (42.4%)
Men	1060 (60.2%)	846 (62.7%)	214 (51.9%)	648 (61.9%)	412 (57.6%)
Age group (years)					
40–54	327 (18.6%)	84 (6.2%)	243 (59.0%)	154 (14.7%)	173 (24.2%)
55–64	436 (24.7%)	293 (21.7%)	143 (34.7%)	253 (24.2%)	183 (25.6%)
65–74	454 (25.8%)	428 (31.7%)	26 (6.3%)	276 (26.4%)	178 (24.9%)
75–84	545 (30.9%)	545 (40.4%)	0 (0.0%)	364 (34.8%)	181 (25.3%)
COVID-19 associated hospital stay	691 (39.2%)	543 (40.2%)	148 (35.9%)	410 (39.2%)	281 (39.3%)
Median (IQR)	10 (4–21)	9 (4–20)	10 (4–26)	9 (4–20)	10 (4–25)
Died ≤30 days after event	369 (20.9%)	301 (22.3%)	68 (16.5%)	208 (19.9%)	161 (22.5%)
Died in study period	458 (26.0%)	383 (28.4%)	75 (18.2%)	258 (24.6%)	200 (28.0%)
Wave					
One	621 (35.2%)	478 (35.4%)	143 (34.7%)	363 (34.7%)	258 (36.1%)
Two	1141 (64.8%)	872 (64.6%)	269 (65.3%)	684 (65.3%)	457 (63.9%)

### Association Between COVID-19 and First Acute Cardiovascular Event

The season-adjusted relative incidence of a first acute cardiovascular event was markedly raised in the first seven days after SARS-CoV-2 with an IR of 7.14 (95% CI 6.06–8.41) and fell sharply to 1.14 (0.92–1.41) by days 29–91 (Table 2). The same pattern, with similar effect estimates, was observed in both raised and low cardiovascular risk profiles (days 1–7: QRISK3 ≥10% 6.97 [5.79–8.39], QRISK3 <10% 7.78 [5.48–11.05], hypertension 6.55 [5.28–8.14], and no hypertension 8.04 [6.26–10.33]). When stratified by wave, the relative incidence of a cardiovascular event in the first seven days post-SARS-CoV-2 was higher, though not significantly, in wave one (6.01 [4.5–8.11]) than wave two (4.19 [3.35–5.23]) (Table 2).

**Table 2:** Season-Adjusted Incidence Ratios for Acute Cardiovascular Events Occurring in Exposed Periods After SARS-CoV-2 Infection by Cardiovascular Risk and COVID-19 Wave

Risk Period	All		QRISK3				Hypertension			
			Raised Risk		Low Risk		Raised Risk		Low Risk	
	N Events	IR (95% CI)	N Events	IR (95% CI)	N Events	IR (95% CI)	N Events	IR (95% CI)	N Events	IR (95% CI)
All										
1–7 days	219	7.14 (6.06–8.41)	171	6.97 (5.79–8.39)	48	7.78 (5.48–11.05)	122	6.55 (5.28–8.14)	97	8.04 (6.26–10.33)
8–14 days	88	3.72 (2.96–4.68)	60	3.22 (2.44–4.24)	28	5.62 (3.68–8.58)	46	3.22 (2.35–4.40)	42	4.50 (3.21–6.31)
15–28 days	64	1.74 (1.33–2.26)	46	1.60 (1.18–2.18)	18	2.22 (1.34–3.68)	42	1.86 (1.34–2.59)	22	1.53 (0.98–2.39)
29–91 days	108	1.14 (0.92–1.41)	76	1.05 (0.81–1.34)	32	1.46 (0.97–2.20)	69	1.17 (0.90–1.53)	39	1.08 (0.76–1.54)
Baseline	714	Ref	548	Ref	166	Ref	433	Ref	281	Ref
Wave 1										
1–7 days	81	6.01 (4.52–7.99)	66	6.13 (4.46–8.43)	15	5.48 (2.91–10.32)	46	5.39 (3.73–7.78)	35	7.15 (4.56–11.23)
8–14 days	20	1.79 (1.11–2.87)	15	1.70 (0.98–2.93)	5	2.09 (0.81–5.44)	9	1.23 (0.62–2.45)	11	2.83 (1.45–5.49)
15–28 days	26	1.36 (0.89–2.07)	20	1.34 (0.83–2.18)	6	1.40 (0.58–3.39)	16	1.28 (0.75–2.19)	10	1.50 (0.75–2.99)
29–91 days	52	0.72 (0.52–1.00)	36	0.65 (0.44–0.95)	16	0.99 (0.53–1.82)	33	0.69 (0.46–1.04)	19	0.78 (0.46–1.34)
Baseline	165	Ref	126	Ref	39	Ref	102	Ref	63	Ref
Wave 2										
1–7 days	138	4.19 (3.35–5.23)	105	3.73 (2.90–4.79)	33	6.62 (4.05–10.83)	76	3.61 (2.69–4.84)	62	5.16 (3.66–7.26)
8–14 days	68	2.69 (2.03–3.57)	45	2.10 (1.50–2.95)	23	5.85 (3.39–10.08)	37	2.35 (1.61–3.42)	31	3.26 (2.13–5.00)
15–28 days	38	0.99 (0.69–1.41)	26	0.80 (0.52–1.22)	12	1.98 (1.00–3.92)	26	1.06 (0.68–1.64)	12	0.85 (0.46–1.59)
29–91 days	56	0.76 (0.55–1.05)	40	0.65 (0.45–0.95)	16	1.36 (0.71–2.63)	36	0.75 (0.50–1.12)	20	0.78 (0.46–1.32)
Baseline	549	Ref	422	Ref	127	Ref	331	Ref	218	Ref

**Figure 3:**
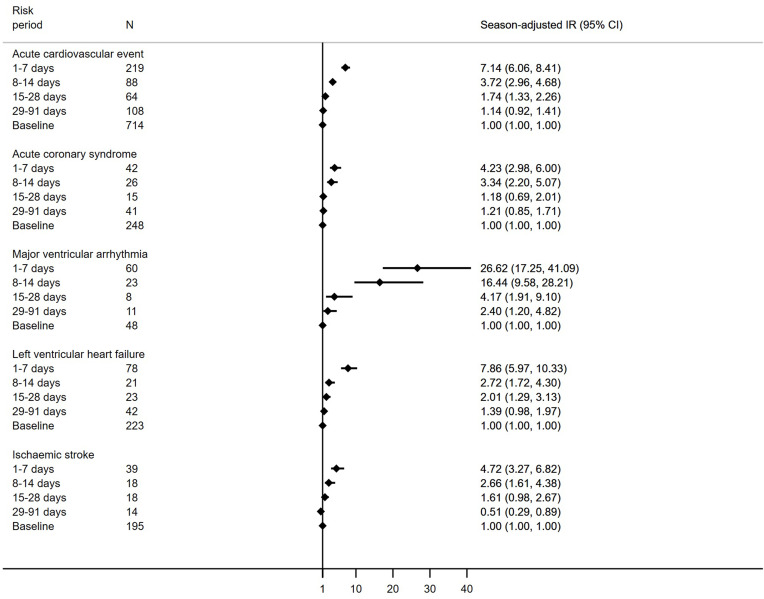
Incidence ratios for first acute cardiovascular events in risk periods following SARS-CoV-2 by cardiovascular event type.

The relative incidence differed by event type; in the first seven days after SARS-CoV-2, the relative incidence was highest for major ventricular arrhythmia (26.62 [17.25–41.09]), although the number of events was small, followed by left ventricular heart failure (7.86 [5.97–10.33]), ischemic stroke (4.72 [3.27–6.82]), and finally ACS (4.23 [2.98–6.00]) (Figure 3).

The IRs were marginally higher in men (days 1–7: 7.90 [6.42–9.72]) than women (days 1–7: 6.10 [4.67–7.97]) and also higher in those aged 40–64 years (days 1–7: 9.61 [7.54–12.26]) than 65–84 years (days 1–7: 5.55 [4.44–6.94]) (Table S1).

### Sensitivity Analysis Using Clinically Reported COVID-19

Redefining infection as clinically reported non-laboratory confirmed COVID-19 yielded 932 individuals with COVID-19 and a first cardiovascular event. Compared to individuals with laboratory-confirmed SARS-CoV-2, a higher proportion of those with clinically reported COVID-19 were women (44.0% vs 39.8%), and a lower proportion were hospitalized due to their COVID-19 (16.1% vs 39.2%) or died within 30 days of their cardiovascular event (16.1% vs 20.9%) or during study follow-up (20.0% vs 26.0%) (Table S2). The IR for first acute cardiovascular event was substantially raised, with a larger magnitude that laboratory-confirmed SARS-CoV-2, in the seven days after diagnosis (10.04 [8.05–12.53]) and although the effect tapered over time, the relative incidence remained raised through all risk windows (days 29–91: 1.76 [1.41–2.20]) (Table S3).

### Sensitivity Analysis Removing People Who Died

After excluding the 369 fatal events, the relative incidence of a first acute cardiovascular event after SARS-CoV-2 decreased compared to when these events were included but remained raised (days 1–7: 4.36 [3.52–5.41]) (Table S4).

## Discussion

In this population-based SCCS study among adults aged 40–84 years with varying cardiovascular risk, we found an increased risk of first acute cardiovascular events in the four weeks after COVID-19, in those with laboratory-confirmed infection or clinically reported illness across all cardiovascular risk profiles. The highest risk was in the first seven days after COVID-19 and the effect tapered over time. Among the individual outcomes of interest, relative incidence post-COVID-19 was highest for major ventricular arrhythmia, followed by left ventricular heart failure, ischemic stroke, and finally ACS. Relative incidence was higher for those aged <65 years, but there is still a low absolute risk of cardiovascular events in this age group. We identified a higher incidence of first acute cardiovascular event following laboratory-confirmed COVID-19 in the first wave of the pandemic than the second.

Our results are consistent with previous SCCS studies investigating COVID-19 associated MI and ischemic stroke using population-based data from Denmark, Sweden and Scotland. Danish National Register data, to mid-July 2020, showed that in the 31 days after COVID-19, there were three- and six-fold increases in the relative incidence of first MI and stroke, respectively, although the study was based on a small sample size with only 17 MI and 44 stroke events.^3^ Swedish EHR data from February to September 2020 illustrated a more than two-fold increase in the relative incidence of first MI in the two weeks after COVID-19 based on 186 events.^2^ However, beyond this time the increase in MI relative incidence was not significant. In comparison, the relative incidence of ischemic stroke remained high in the month after COVID-19, although it was only based on 254 events. A SCCS study of Scottish data with 1449 individuals, using a study period of March 2018 to October 2020, evaluated the risk of MI, ischemic stroke, pulmonary embolism and deep vein thrombosis. The study identified a 12-fold increase in relative incidence in the first seven days after COVID-19.^4^

Cardiovascular complications are not uniquely triggered by COVID-19. Other acute respiratory infections including GP-diagnosed acute respiratory infections,^23^ influenza-like illnesses,^24^ laboratory-confirmed respiratory virus infections and *Streptococcus pneumoniae*^25^ are also associated with a transient increase in MI and stroke risk. We recently demonstrated that underlying cardiovascular risk impacts the likelihood of first acute cardiovascular event after an acute respiratory infection; those with raised cardiovascular risk had a 2–3 times higher risk of cardiovascular complications after ARI.^26^ Nevertheless, individuals at raised cardiovascular risk but without existing CVD are not traditionally considered high risk for severe outcomes after respiratory infections.^27^

SARS-CoV-2, like other ARI causative agents gives rise to inflammatory, thrombotic and microvascular dysfunction events which can lead to new cardiovascular complications.^28^ SARS-CoV-2 additionally has greater systemic effect. Angiotensin-converting enzyme 2 is a surface protein that is key to SARS-CoV-2 cell entry but is also part of the renin-angiotensin-aldosterone system, which is important for normal functioning of the cardiovascular system, so likely to play a role in the cardiovascular effects of the virus.^29^ Numerous cardiovascular risk factors, including obesity, hypertension and diabetes, have been implicated in the interaction between SARS-CoV-2 and cardiovascular complications^30^ and may give rise to more severe disease and increase COVID-19 associated cardiovascular complications.^28^

Prevention and management of cardiovascular risk factors, along with COVID-19 itself, should be sustained and prioritised. The results generated by our study, and those of previous studies, show a spectrum of acute cardiovascular complications associated with COVID-19, with the timing of risk varying for different outcomes. Further understanding of longer-term cardiovascular outcomes, including the cardiovascular manifestations and mechanisms of post-COVID-19 syndrome, is an important focus for future research.^30^

We included a large study population using primary and secondary care linked data generalizable to the population of England in terms of age, sex and deprivation.^31^ However, the use of the SCCS method means only individuals with COVID-19 who had an acute cardiovascular event were included, limiting generalizability to those at risk of cardiovascular disease. A strength of SCCS is its implicit control for fixed between-person confounding. We took steps to ensure our study did not violate SCCS assumptions with first cardiovascular events included, excluded the 14 days before COVID-19 from analysis, and repeated analysis without fatal events to confirm the validity of main results.

At the start of the pandemic, laboratory testing for SARS-CoV-2 was targeted towards individuals with a clinical need. This will have limited the number of individuals entering our study if their COVID-19 illness was early in the pandemic. We included a study population with clinically reported COVID-19 to account for this data bias. We unexpectedly identified a much higher relative incidence of major ventricular arrhythmia (although there was a small number of events) compared to other cardiovascular complications. This finding may be due to differential detection of outcomes, with arrhythmias occurring, for example, as a complication of an earlier undetected cardiovascular event. Furthermore, our study used routinely-collected healthcare data which does not permit the inclusion of detailed clinical data such as troponin or B-type natriuretic peptide levels. Evaluating any association between COVID-19 and exacerbation of chronic vascular disease eg chronic limb ischaemia was beyond the scope of our study, which focused on the first acute cardiovascular events. To avoid the impact of major SARS-CoV-2 variants of concern or vaccination, we limited our study to 2020. However, improved treatment options were in use by wave two of the pandemic, which may explain the higher relative incidence identified in wave one compared to wave two.

In conclusion, we showed an elevated risk of a range of acute cardiovascular events in the days and weeks after SARS-CoV-2 infection in both waves one and two of the pandemic. Prevention of COVID-19 is important to avert cardiovascular complications. Prevention and management of cardiovascular risk factors, which were negatively impacted by the pandemic,^32^ will also alleviate the burden of COVID-19 associated cardiovascular events.

## Data Sharing Statement

The data used for this study were obtained from the CPRD. All CPRD data are available via an application to the Independent Scientific Advisory Committee (see https://www.cprd.com/Data-access). Data acquisition is associated with a fee and data protection requirements. This study is supported by code lists used to define each health condition, which have been made openly available at https://doi.org/10.17037/DATA.00002763. Our data management and analysis computer code is available via GitHub at https://github.com/jenAdavidson/cvrisk_covid_sccs. All code is shared without investigator support.
